# Transcriptome Analysis to Understand Salt Stress Regulation Mechanism of *Chromohalobacter salexigens* ANJ207

**DOI:** 10.3389/fmicb.2022.909276

**Published:** 2022-06-30

**Authors:** Alok Kumar Srivastava, Ruchi Srivastava, Anjney Sharma, Akhilendra Pratap Bharati, Jagriti Yadav, Alok Kumar Singh, Praveen Kumar Tiwari, Anchal Kumar Srivatava, Hillol Chakdar, Prem Lal Kashyap, Anil Kumar Saxena

**Affiliations:** ^1^Indian Council of Agricultural Research-National Bureau of Agriculturally Important Microorganisms, Mau, India; ^2^Department of Life Science and Biotechnology, Chhatrapati Shahu Ji Maharaj University, Kanpur, India; ^3^Indian Council of Agricultural Research-Indian Institute of Wheat and Barley Research, Karnal, India

**Keywords:** *Chromohalobacter salexigens*, q-RNA sequencing, salt stress, osmoregulation, betain

## Abstract

Soil salinity is one of the major global issues affecting soil quality and agricultural productivity. The plant growth-promoting halophilic bacteria that can thrive in regions of high salt (NaCl) concentration have the ability to promote the growth of plants in salty environments. In this study, attempts have been made to understand the salinity adaptation of plant growth-promoting moderately halophilic bacteria *Chromohalobacter salexigens* ANJ207 at the genetic level through transcriptome analysis. In order to identify the stress-responsive genes, the transcriptome sequencing of *C. salexigens* ANJ207 under different salt concentrations was carried out. Among the 8,936 transcripts obtained, 93 were upregulated while 1,149 were downregulated when the NaCl concentration was increased from 5 to 10%. At 10% NaCl concentration, genes coding for lactate dehydrogenase, catalase, and OsmC-like protein were upregulated. On the other hand, when salinity was increased from 10 to 25%, 1,954 genes were upregulated, while 1,287 were downregulated. At 25% NaCl, genes coding for PNPase, potassium transporter, aconitase, excinuclease subunit ABC, and transposase were found to be upregulated. The quantitative real-time PCR analysis showed an increase in the transcript of genes related to the biosynthesis of glycine betaine coline genes (gbcA, gbcB, and L-pro) and in the transcript of genes related to the uptake of glycine betaine (OpuAC, OpuAA, and OpuAB). The transcription of the genes involved in the biosynthesis of L-hydroxyproline (proD and proS) and one stress response proteolysis gene for periplasmic membrane stress sensing (serP) were also found to be increased. The presence of genes for various compatible solutes and their increase in expression at the high salt concentration indicated that a coordinated contribution by various compatible solutes might be responsible for salinity adaptation in ANJ207. The investigation provides new insights into the functional roles of various genes involved in salt stress tolerance and oxidative stress tolerance produced by high salt concentration in ANJ207 and further support the notion regarding the utilization of bacterium and their gene(s) in ameliorating salinity problem in agriculture.

## Introduction

Salinity is one of the major threats to crop production all over the globe (Sharma et al., 2015; Kushwaha et al., 2020; Kojonna et al., 2022). It has been reported that about 20% of total cultivated and 33% of irrigated agricultural lands in the world are affected by salinity and sodicity problems, which resulted in the reduction of the average yield of major food grain crops by >50% (Shrivastava and Kumar, 2015; Kashyap et al., 2017; Sharma et al., 2019; Daba and Qureshi, 2021). At present, salinity is reported as a major problem in over 100 countries, and no continent is untouched by this malady (Parihar et al., 2015; Shahid et al., 2018). The countries where salt-affected soils exist at a large scale include, but are not restricted to, Australia, Bangladesh, China, Egypt, India, Iran, Iraq, Mexico, Pakistan, Syria, Turkey, the former USSR, and the United States (Chhabra, 2022). In India, most states like Gujarat, Uttar Pradesh, Maharashtra, West Bengal, and Rajasthan are struggling with the same issue (Sharma and Singh, 2017; Srivastava et al., 2019a,b). This affects most of the stages of crop development and production such as germination, plant growth, flowering, fruiting, seed setting, and yield (Kashyap et al., 2020). In this connection, several attempts have been made to increase the salt tolerance of crops with techniques ranging from selection within species, hybridization with wild relatives, the use of cell culture, and the use of genes to develop transgenic plants which can overcome salt stress (Sanghera et al., 2011; Kotula et al., 2020). Unfortunately, these approaches are time-consuming and economically unviable. Application of halophilic and halotolerant bacteria that promote plant growth can be used as one of the cheap, environmentally friendly, and promising alternatives to alleviate the toxic effects of salinity (Alexander et al., 2020; Orhan, 2021).

Halophilic bacteria are endowed with a unique inherent character of salt tolerance and adopt diverse types of osmoadaptation mechanisms, which confers them considerable importance these days due to their utilization in the agriculture, food, and fermentation industries (O’Byrne and Booth, 2002; Vaidya et al., 2018). The intracellular accumulation of the small organic osmolytes is a more common strategy to cope with the osmotic stress produced by the presence of high salt concentrations in the extracellular environment. These osmolytes have been reported to protect the plant cells from the high salt concentrations and also function as osmoprotectants (Slama et al., 2015). These are also termed compatible solutes, as they provide osmotic balance without interfering with the cell function and proper folding of the protein. It is worth mentioning that microorganisms have evolved with a variety of transporters and efflux systems to maintain osmolarity (Kashyap et al., 2016; Hoffmann and Bremer, 2017). There are several compounds, for example, sugar molecules (sucrose and trehalose), polyols (glycerol, glucosylglycerol, arabitols, etc.), amino acids (proline, hydroxyproline, alanine, glycine, glutamate derivatives, etc.), quaternary amines (betain, choline, etc.), and ectoine and its derivatives, that act as osmoprotectants (Patel et al., 2018; Salvador et al., 2018; Khatibi et al., 2019; Wiesenthal et al., 2019). These organic molecules can be either synthesized in the cell or can be transported from the extracellular environment. Most of the molecules are accumulated in the cell because of their *de novo* synthesis by specific biosynthetic pathways, but the uptake of the osmoprotectant from the external environment is energetically preferred over *de novo* synthesis (Roberts, 2005; Vargas et al., 2008).

*Chromohalobacter salexigens* is a halophilic γ-proteobacterium that grows optimally in high salt concentrations (Arahal et al., 2001; Srivastava et al., 2019a). Being a highly salt-tolerant microorganism, several groups have used this as a model organism to study osmoadaptation in prokaryotes (Vargas et al., 2008). *C. salexigens* mainly adopt two strategies for survival under osmotic stress: first is either *de novo* synthesis of the osmoprotectants or uptake from the environment and second is the enhancement in membrane adaptation *via* the synthesis of the membrane cardiolipin and cyclopropane fatty acids (Vargas et al., 2008). *C. salexigens* has also been reported to synthesize ectoine and β-hydroxyectoine as the main osmoprotectants in the absence of the main compatible solutes betain in the external environment (García-Estepa et al., 2006; Salvador et al., 2018). The osmoprotectants accumulated in response to increasing salinity act as protecting agents for cells and their organelles. *C. salexigens* ANJ207 exhibits PGP traits, that is, siderophore production and Zn, P, and K solubilization, at higher salt concentrations and has shown promising results in wheat and rice (unpublished results). Although the beneficial plant growth-promoting effects of *C. salexigens* under salt stress have been observed by other workers (Anbumalar and Ashokumar, 2016; Elsakhawy et al., 2019), the underlying molecular mechanisms and the genes responsible for salt tolerance, along with their gene expression levels, need to be identified to optimize the field applications of *C. salexigens* in agricultural and allied sectors. At present, limited information is available for understanding the dynamics of complex salt interactions and the genes responsible for salt tolerance in *C. salexigens*. Therefore, in the present study, attempts have been made to fill this research gap by exploring the salinity adaptation of *C. salexigens* ANJ207 at the genetic level through transcriptome analysis using next-generation sequencing and qRT-PCR to obtain new insights into the understanding of the adaptation of *C. salexigens* ANJ207 to salt stress. We reported that the number of upregulated genes was positively correlated with salt concentration. Moreover, higher salt concentration not only induces the genes related to osmolarity regulation but also induces the genes related to protein folding and oxidative stress. We also confirmed the expression of the genes for glycine betain and proline biosynthesis, as well as the transporters using the qRT-PCR. The qRT-PCR results confirm the validity of the transcriptome analysis. These findings will further help to unravel the complex biological mechanisms regarding osmotic stress adaptation and various mechanisms involved in the production of secondary metabolites under saline conditions. Harnessing the potential of *C. salexigens* ANJ207 and its secondary metabolites for the development of novel bioinoculant can be one of the prospective solutions to overcome the soil salinity problem in the near future.

## Materials and Methods

### Bacterial Strains and Growth Conditions

*Chromohalobacter salexigens* is one among the nine known species in the genus *Chromohalobacter* belonging to the family Halomonadaceae. *Chromohalobacter salexigens* ANJ207 was isolated from the salt crystals deposited in the pipelines of the Indian Council of Agricultural Research-National Bureau of Agriculturally Important Microorganisms (25.8982° N and 83.4891° E) and grew profusely in the presence of 30% NaCl (Srivastava et al., 2019a).

### RNA Extraction, Library Construction, and RNA Sequencing

To investigate the transcriptional response of *C. salexigens* ANJ207 to osmotic stress, three RNA-Seq libraries were generated from three different conditions: low salinity (5% NaCl), optimal salinity (10% NaCl), and high salinity (25% NaCl). Cell samples were collected during the exponential phase when cultures reached enough biomass to assure isolation of 100–500 ng of mRNA for RNA-Seq library construction. Total RNA was isolated from *Chromohalobacter salexigens* ANJ207 grown at different concentrations of salt [NaCl 5% (C1), 10% (C2), and 25% (C3)] using the manufacturer’s protocol of GeneJET RNA purification kit with slight modification as described elsewhere (Srivastava et al., 2021). Two biological replicates were used for transcriptome analysis. The *de novo* transcriptome sequencing was performed using the Illumina HiSeq 2500 platform in the paired-end module (Liu et al., 2014). The raw fastq files were processed before performing assembly. Prior to the assembly, base trimming, removal of adapter sequences, and filtering out reads with an average quality score of less than 30 were performed in every paired-end read. Further, the rRNAs were removed based on the reference data from the SILVA database (Quast et al., 2012). The cleaned reads were aligned to the assembled transcriptome (length ≥ 200 bp) using the Bowtie2 program (Langmead and Salzberg, 2012). The cleaned RNA-Seq reads from the libraries were normalized and subjected to *de novo* transcriptome assembly using Trinity (Huang et al., 2016). Normalization was performed using the variance analysis package of the EdgeR program (Robinson et al., 2010). The assembled transcripts were annotated using BLASTX against the non-redundant nucleotide databases. Bioconductor EdgeR Package was used for the differential gene expression analysis. The abundance of all genes was calculated using particularly mapped reads by the fragments per kilobase of transcript per million fragments mapped (FPKM) through RPKM functions of EdgeR. To determine the threshold *p*-value in multiple tests, Benjamin and Hochberg’s method of false discovery rate (FDR) (Benjamini and Hochberg, 1995) was used. The *p*-value cutoff was kept at < 0.05.

### Quantitative PCR Analysis for Validation of RNA-Seq Data

A single colony of *Chromohalobacter salexigens* ANJ207 was inoculated into 100 ml of nutrient broth medium containing 5% NaCl and grown overnight at 32°C and 150 rpm. The secondary inoculation was done in triplicate using 1% overnight grown primary culture in 100-ml conical flasks containing 50 ml of sterile nutrient broth and incubated at different concentrations of NaCl (5, 10, and 25%) at 150 rpm for 12 h. Total RNA was isolated from *Chromohalobacter salexigens* ANJ207 grown at different concentrations of NaCl (5, 10, and 25%) using the manufacturer’s protocol of GeneJET RNA purification kit with slight modifications (Srivastava et al., 2021). RNA intensity and purity were checked by qualitative (1.2% formaldehyde agarose gel electrophoresis) and quantitative analysis (using Nanodrop). The cDNA was synthesized using 2 μg of RNA from each sample of *Chromohalobacter salexigens* ANJ207 by using an iScript cDNA synthesis kit with oligo (dT) and random hexamer primers.

The quality of the cDNA was checked by simple amplification of the 16S rRNA gene. Each cDNA sample was diluted in nuclease-free miliQ to obtain the concentration of 100 ng/μl for the qRT-PCR experiment, and 16S rRNA RT primers were used as endogenous control. SSO Fast EvaGreen Supermix (Biorad) was used. For real-time PCR, initial heat activation at 95°C for 5 min, followed by 40 cycles of amplification by a three-step cycling protocol (denaturation at 95°C for 30 s, annealing at 55°C for 30 s, and extension at 65°C for 45 s) was done. Melting curve analysis was performed by heating the plate at 95°C for 30 s, incubating at 65°C for 30 s, and then heating to 95°C for 30 s. The sample was performed in triplicates. G8830A AriaMx Real-Time PCR system from Agilent was used for the experimentation, and the results were analyzed by the Agilent AriaMx software version 1.5.

## Results and Discussion

### *Chromohalobacter salexigens* ANJ207 Growth Stimulation by a Broad Range of Salt Concentration

The strain was isolated from the salt crystals deposited in the pipelines of the Indian Council of Agricultural Research National Bureau of Agriculturally Important Microorganism (ICAR-NBAIM), Kushmaur, Mau (Srivastava et al., 2019a). The salt crystals were added to the nutrient broth with 20% NaCl (wt/vol) and incubated at 28°C for 72 h. The broth was then serially diluted till 10^–5^ dilution, and 100 μl aliquots from the dilutions 10^–2^ to 10^–4^ were spread on nutrient agar (NA) supplemented with 2.5–35% NaCl.

**FIGURE 1:**
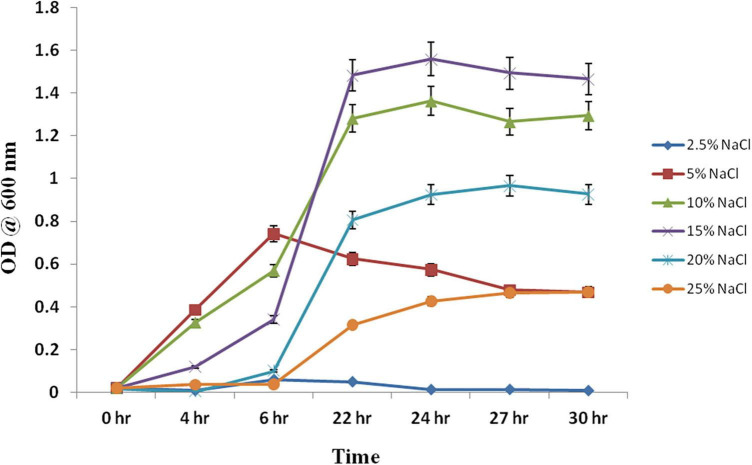
Growth kinetics at different concentrations of the NaCl. *Chromohalobacter salexigens* ANJ207 was grown in the flask with the initial equal cell concentration at different concentrations of salt. The growth was monitored and OD_600_ was calculated at different time intervals. OD_600_ was plotted against different time intervals.

*Chromohalobacter salexigens* ANJ207 was grown at different regimes of salt (NaCl) ranging from 2.5% to 25%, and the samples were collected at different time intervals and a growth curve was prepared (Figure 1). Based on the growth curve analysis at different salt concentrations, we concluded that the strain was not able to grow below 2.5% salt concentration. This result indicated that ANJ207 requires at least 2.5% NaCl for growth, which was in line with earlier published reports, where a minimal salt requirement is reported as an essential component for the growth initiation of a moderate halophile *Chromohalobacter salexigens* (O’Connor and Csonka, 2003). Initially, at 5% NaCl concentration ANJ207 grows efficiently, but after some time the growth was retarded. The salt concentration of 10–15% was considered to be an optimal concentration where it grows efficiently with a half-generation time of 3–4 h, respectively. After that, at 20 and 25% salt concentration, the growth was very slow with a mean generation time of 5–10 h^–1^, respectively (Table 1). These results are consistent with earlier studies, where halophilic bacteria (*Halobacillus halophilus*, *Halobacillus litoralis*, *Bacillus halophilus*, *Marinococcus halophilus*, and *Saliiococcus hispanicus*) were documented to grow optimally at NaCl concentrations ranging from 10 to 15% (Sarwar et al., 2015). In addition, plant growth-promoting halophilic bacteria, that is, *Halomonas pacifca*, *H. stenophila*, *Bacillus haynesii*, *B. licheniformis*, and *Oceanobacillus aidingensis*, were also reported to grow optimally in the media containing 10–15% NaCl concentration, although they were able to tolerate up to 25% NaCl concentration with restricted growth (Reang et al., 2022).

**TABLE 1:** Growth statistics of *Chromohalobacter salexigens* ANJ207.

NaCl conc (%)	Growth rate constant (h^–1^)	Mean generation time (h)
2.5	0.002	500
5	0.048	20.833
10	0.232	4.310
15	0.296	3.378
20	0.195	5.128
25	0.098	10.204

### RNA-Seq Analysis Reveals Salt Stress-Driven Expression of *Chromohalobacter salexigens* ANJ207 Transcripts

RNA-Seq analysis at different salt concentrations (5, 10, and 25% termed as C1, C2, and C3, respectively) was performed to investigate the transcriptome changes in salt stress. An average of 38,857,852, 35,751,018, and 44,695,124 million raw paired-end reads were obtained from C1, C2, and C3 samples, respectively and low-quality mapped reads were evaluated and eliminated (Supplementary Table 1). After pre-processing the data, an average of 37,440,398, 34,429,792, and 42,932,742 million cleaned paired-end reads were obtained for C1, C2, and C3, respectively (Supplementary Table 2). The RNA-Seq reads from three libraries were combined together using the Trinity software, and the final *de novo* transcriptome was assembled having 8,936 transcripts. The average calculated length of the transcript was found to be ∼1,366.71 bp with the N_50_ of 4,109 (Supplementary Table 3). Subsequently, the annotation of the assembled transcripts was done using the BLASTX. Non-redundant (NR) nucleotide databases were used for the blast search. Out of the 8,936 transcripts, 8,649 transcripts had at least one significant hit, which was identified by a BLASTX search. The cleaned reads were aligned to the assembled transcriptome (length ≥ 200 bp) using the Bowtie2 program (Langmead and Salzberg, 2012). The alignment summary is provided in Supplementary Table 4. The expression level of the sequencing data was evaluated using FPKM values obtained through RPKM functions in EdgeR (Abbas-Aghababazadeh et al., 2018; Chen et al., 2020). The distribution statistics of the FPKM values are listed in Supplementary Figure 1. The figure showed that most of the genes were between 1 and 10 expression level categories.

Because of the differences in the length of the genes and the variation in the library size in each sample, the deviation can be seen in the RNA-Seq analysis. So it is very important to normalize the data for the removal of the differences in statistical deviation that can distort the sequencing analysis (Risso et al., 2011; Hansen et al., 2012). The data were normalized using the variance analysis package EdgeR program, followed by the calculation of the *p*-value (Anders and Huber, 2010). The *p*-value determines the statistical significance of the number of reads per gene in the biological samples (Tan et al., 2003). Using the EdgeR software, the number of the reads for each transcript can be mapped for the differential gene expression analysis. The abundance of a particular transcript in different samples of the RNA-Seq was revealed by the counts per million (CPM) value. The CPM represents the expression value of the transcript. A FDR cutoff for *p*-value was applied (<0.05) to select 1,242, 3,123, and 3,241 differentially expressed genes (DEGs) (Table 2).

**TABLE 2:** List of the number of transcripts upregulated or downregulated in different differential expression combinations obtained from the EdgeR program.

Deseq Combination	Upregulated transcripts	Downregulated transcripts	Transcripts with no significance
C1 vs. C2	93	1149	7694
C1 vs. C3	1418	1705	5813
C2 vs. C3	1954	1287	5695

**FIGURE 2:**
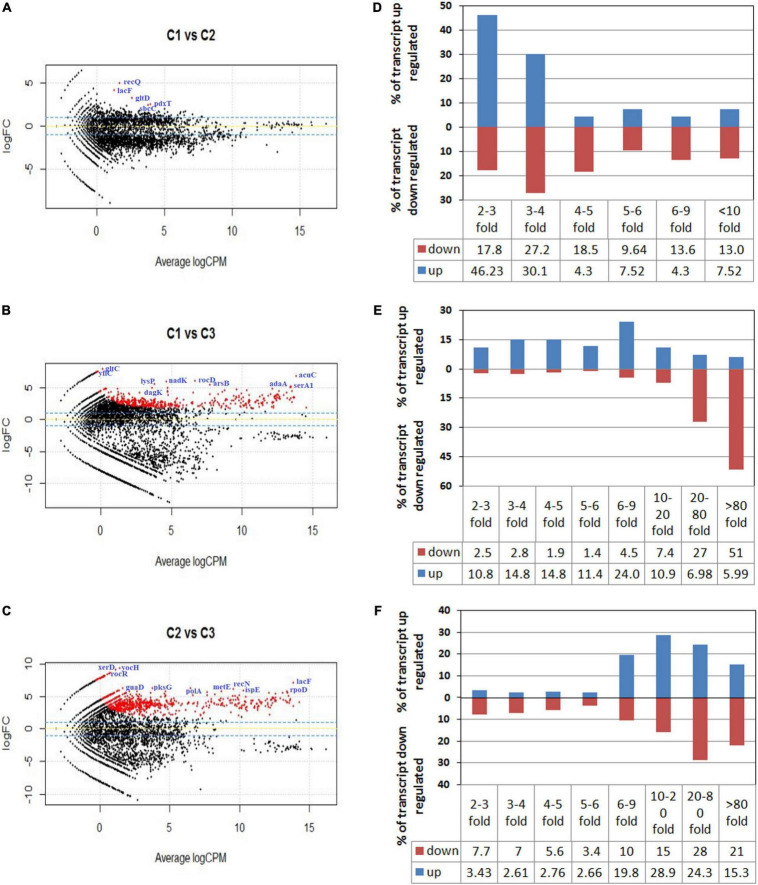
MA plot for differential expression analysis generated by EdgeR. **(A–C)** Represent the MA plot of the differentially expressed genes (DEGs) when the RNA sequencing data of samples C1 (5% NaCl), C2 (10% NaCl), and C3 (25% NaCl) were compared with each other. Plot A represents the DEGs of C1 vs. C2, while **(B,C)** plots represent the DEGs of C1 vs. C3 and C2 vs. C3, respectively. Each gene is represented by a black dot. The blue lines represent the log FC ± 1.0 threshold, and the yellow line indicates a log ratio of zero. The dots in the plus direction represent the upregulation and in the minus direction represent the downregulated genes. **(D–F)** Represent the comparisons of the DEGs of C1 vs. C2, C1 vs. C3, and C2 vs. C3 samples. They represent the percentage of the upregulated and downregulated transcripts in different fold change categories.

**FIGURE 3:**
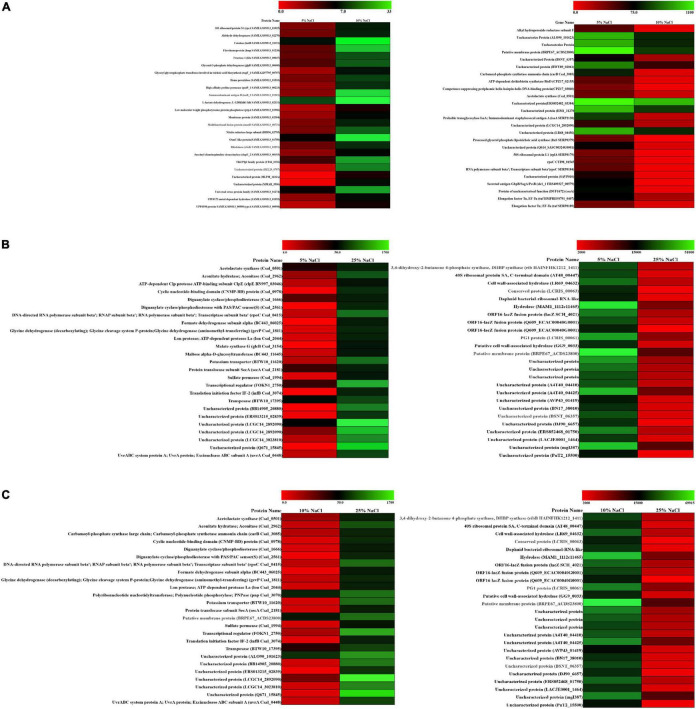
Differential expression of gene (DEG) analysis of ANJ207. **(A)** Heatmap of the C1 vs. C2 upregulated and downregulated genes, **(B)** heatmap of the C1 vs. C3 upregulated and downregulated genes, and **(C)** heatmap of the C2 vs. C3 upregulated and downregulated genes. Red color indicates no expression while green indicates the highest expression.

To graphically differentiate the DEGs, an MA plot was drawn between log fold change in expression and average log CPM (Figures 2A–C). Each gene is represented by a black dot. The blue lines represent the log FC ± 1.0 threshold, and the yellow line indicates a log ratio of zero. The dots in the plus direction represent the upregulation and in the minus direction represent the downregulated genes. Figure 2A clearly indicates that at a low concentration of the salt (C1), the majority of the genes displayed downregulation. On the other hand, upregulation in the level of gene expression was noticed in the cases of C2 and C3 with a rise in salt concentration (Figures 2B,C). The genes upregulated and downregulated at each salt concentration C1 (5%), C2 (10%), and C3 (25%) were also compared. The result shows that from C1 to C2 only 93 genes were upregulated. Further, it has been noticed that 1,149 genes were downregulated and in 7,694 genes no significant change was observed. Similarly, if we compare C2 vs. C3, 1,954 genes were upregulated, 1,287 genes were downregulated, and 5,695 genes showed no significant change (Table 2). These results clearly indicate that at lower concentrations of NaCl, most of the genes were in downregulation mode. If we compare C2 vs. C3 at a higher concentration, the number of genes upregulated was high when compared to the lower concentration, while the number of genes downregulated was approximately the same (1,287 transcripts) when compared to the lower concentration (1,149 transcripts). We calculated the percentage of genes upregulated or downregulated at different fold changes (Figures 2D–F). The comparison between the C1 and C2 samples reveals that 76% of the upregulated transcripts and 45% of the downregulated transcripts fall within the range of 2–4-fold change (Figure 2D). Similarly, the comparison between the C1 and C3 samples showed only 25% of the upregulated transcripts and 5% of the downregulated transcripts within the range of 2–4-fold change (Figure 2E). Around 24% of the upregulated transcripts come within the range of 6–9-fold change, while more than 50% of the downregulated transcript fall within more than 80-fold change in expression (Figure 2E). The comparison between the C2 and C3 samples represents the irregularity in the differential gene expression. We found around 68% of the upregulated transcript within the range of 10–80-fold change in expression and 64% of the downregulated transcripts within the range of more than 10–80-fold change (Figure 2F). The comparative figures of the top 25 upregulated and downregulated genes in C1, C2, and C3 samples were represented in the form of a heatmap (Figure 3).

### Gene Ontology and Annotation Analysis Reflect Shift in the Expression of the Genes Related to the Osmolarity Balance

The assembled transcripts were annotated using an in-house pipeline. First, a comparison with the UniProt database using the BLASTX program and then the ontology annotation followed by organism annotation were done. The assembled transcripts were compared with the UniProt database using the BLASTX program with an *E*-value cutoff of 10^–3^. The best BLASTX hit based on query coverage, identity, similarity score, and description of each transcript was filtered out using our in-house pipeline. The BLASTX summary is provided in Supplementary Table 5. The *E*-value and similarity score distribution of BLASTX hits are provided in Supplementary Figure 2. The gene ontology (GO) terms molecular function (MF), cellular component (CC), and biological process (BP) for transcripts were also mapped against the latest GO database.

**FIGURE 4:**
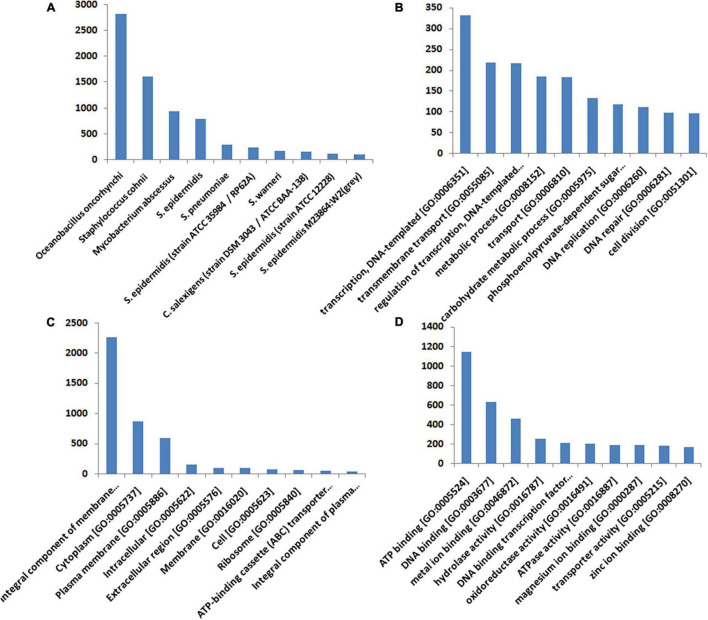
Annotation and the gene ontology of DEGs. The assembled transcripts were compared with the UniProt database using the BLASTX program with an *E*-value cutoff of 10^– 3^. The best BLASTX hit based on query coverage, identity, similarity score, and description of each transcript was filtered out using an in-house pipeline. Based on the BLASTX summary and *E*-value and similarity score distribution of BLASTX hits, the annotation was done. **(A)** Shows the distribution of the top 10 organisms corresponding to the best BLASTX hits. **(B–D)** Represent the top 10 categories of each gene ontology (GO) in terms of molecular function, cellular component, and biological process, respectively. The transcripts were also mapped against the latest GO database.

More than 37% of the significant hits came from the *Staphylococcus* sp. [*S epidermidis* (2.4%), *S. warneri* (1.8%), *S. epidermidis* (11.8%), *S. pneumoniae* (3.23), and *S. cohnii* (18.65%)]. *Oceanobacillus oncorhynchi* contributes to 32.48% of the transcripts, while *C. salexigens* contributes only 1.65% of the transcript (Figure 4A). To classify the function of the assembled transcripts, a GO assignment was carried out. In the “Biological Process” category, the top 10 GO terms represented in the figure include transcription DNA-templated [GO: 0006351], transmembrane transport [GO: 0055085], regulation of transcription, DNA-templated [GO: 0006355], metabolic process [GO: 0008152], transport [GO: 0006810], carbohydrate metabolic process [GO: 0005975], phosphoenolpyruvate-dependent sugar phosphotransferase system [GO: 0009401], DNA replication [GO: 0006260], DNA repair [GO: 0006281], and cell division [GO: 0051301] (Figure 4B). In the “Cellular Component” category, the top 10 GO terms were integral component of membrane [GO: 0016021], cytoplasm [GO: 0005737], plasma membrane [GO: 0005886], intracellular [GO: 0005622], extracellular region [GO: 0005576], membrane [GO: 0016020], cell [GO: 0005623], ribosome [GO: 0005840], ATP-binding cassette (ABC) transporter complex [GO: 0043190] and integral component of plasma membrane [GO: 0005887] (Figure 4C). In the “Molecular Function” category, the top 10 GO terms were ATP binding [GO: 0005524], DNA binding [GO: 0003677], metal ion binding [GO: 0046872], hydrolase activity [GO: 0016787], DNA binding transcription factor activity [GO: 0003700], oxidoreductase activity [GO: 0016491], ATPase activity [GO: 0016887], magnesium ion binding [GO: 0000287], transporter activity [GO: 0005215], and zinc ion binding [GO: 0008270] (Figure 4D). The results of the GO analysis represent a shift in the expression of the genes mostly involved in oxidative stress, stress damage response, and transporters related to the osmolarity balance.

### Differentially Expressed Genes Involved in Adaption to Salt Stress

**FIGURE 5:**
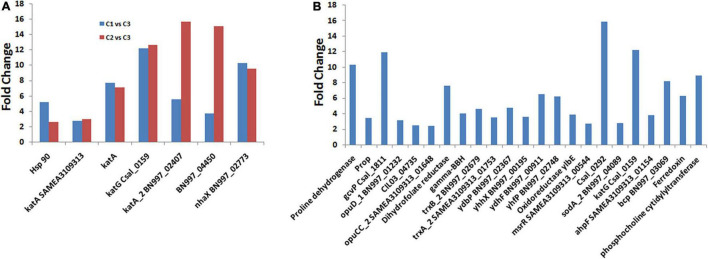
Differentially expressed genes (DEGs) involved in adaption to osmotic and oxidative stress. **(A)** Represents the comparison of the fold change in some of the transcripts involved in salt stress between the C1 vs. C3 and C2 vs. C3, and **(B)** represents the fold change of the DEGs involved in osmotic and oxidative stress conditions.

Salinity is responsible for different types of stresses, like osmotic stress, ionic stress, oxidative stress, and hormonal imbalance, in microorganisms (Etesami and Glick, 2020; Mahmood et al., 2022). Besides the osmotic stress, it also induces heat shock stress, leading to the misfolding of proteins (Roncarati and Scarlato, 2017). In *C. salexigens* ANJ 207, we observed that only few transcripts were upregulated when C1 and C2 groups were compared, but we observed more genes when the salt concentration was increased. We have listed some of the genes in Supplementary Table 6. We also calculated the fold change in the expression of these genes when the cells were shifted from C1 to C2 condition and from C2 to C3 condition (Figure 5A and Supplementary Table 6). Catalase is one of the proteins that is expressed during oxidative stress and was also found to be increased by more than 2–16-fold in transcriptome sequencing analysis. We also observed more than one copy of the catalase enzyme, which showed variation in their expression level at different salt concentrations. Besides the osmotic stress, we also observed an increase in the expression of the heat shock protein (HSP), which was reported to show more than a five fold increase in the expression in our transcriptome sequencing data. We also observed more than a 10-fold increase in a stress-responsive gene homologous to NhaX reported in the *Bacillus subtilis* (Gene ID: 939286).

Microorganisms employ diverse types of adaptation mechanisms to deal with osmotic stress (Chen et al., 2017; Gunde-Cimerman et al., 2018). It mainly depends on either the *de novo* synthesis or the uptake from the environment (Roberts, 2005). The tripartite ATP-dependent and ATP-independent transporter involved in the specific uptake of salinity-compatible solutes was initially described in *H. elongate* (Schweikhard et al., 2010). Additionally, we also observed the presence of diaminobutyrate-2-oxoglutarate transaminase (EctB) in the ectoine biosynthesis in coordination with the *EctA* and *EctC* genes. The presence of the recycling pathway finely adjusts the internal concentration of ectoines in response to the osmolarity changes (Schwibbert et al., 2011). Overall, we did not observe a very significant increase in the ectoine or hydroxyectoine synthesis, as had been already observed in other *Chromohalobacter* in osmotic stress conditions (Vargas et al., 2008; Czech et al., 2018).

RNA-Seq analysis performed in the present study revealed the induction of genes encoding ATP-binding-cassette (ABC) transport systems for betaine and choline as well as the tripartite ATP-independent transport system with a rise in salinity levels (Supplementary Table 7), which is in conformity with the earlier published literature (Gregory and Boyd, 2021). At high salinity, overexpression of orthologous genes for ProP, OpuD, and ABC transporters and genes for proline dehydrogenase and glycine dehydrogenase was already been observed in *Chromohalobacter* (Chen et al., 2010; Malek et al., 2011). Here, we identified more than 10-fold upregulation in the transcripts of proline dehydrogenase and glycine dehydrogenase (Figure 5B), but did not observe the overexpression of ectoine. The explanation of this observation may be that the synthesis of the ectoines was suppressed by the extracellular betaine or its precursor choline (Calderón et al., 2004; Vargas et al., 2006). Along with the glycine betaine transporter, we also observed overexpression of the choline transporters. These findings suggest that the uptake of the osmoprotectants from the environment is preferred over *de novo* synthesis under osmotic stress, as it is energetically cheaper to the cell (Vargas et al., 2008).

Besides the osmotic stress, the high salt condition also induced oxidative stress due to the generation of reactive oxygen species. At low salinity, genes encoding thioredoxin reductase (csal2959), oxidoreductase (YhhX, YdhF, and YlbE), quinine oxidoreductase (YhfP), and methionine sulfoxide reductases (msrR SAMEA3109313_00544) were induced (Figure 5B and Supplementary Table 7), which are among the essential components of protein repair system (Ezraty et al., 2017). High salinity induced the expression of detoxifying enzymatic mechanisms, for instance, a catalase orthologous to KatG (csal_0159) was induced more than 10 times. In addition, iron superoxide dismutase, the peroxidase OsmC, a putative peroxiredoxin, and an alkyl-hydroperoxide reductase were induced (Figure 5B and Supplementary Table 6). In addition, a different set of genes related to the maintenance of redox balance were also affected, which led to more than 15 times upregulation of the gene related to glutathione metabolism (i.e., 4-hydroxyphenylpyruvate dioxygenase, Csal_0292). The genes related to oxidative damage repair were overexpressed, such as glutaredoxin, ferredoxin, KatE, catalase, alkyl hydroperoxide reductase, superoxide dismutase, peroxidases, peroxiredoxin, etc. (Supplementary Table 7). The genes related to the detoxification of the reactive oxygen species produced by nitrogen-containing molecules, such as NorD and NorM, were also induced (Supplementary Table 7). Similar observations regarding the induction of the expression of genes encoding direct reactive oxygen species (RO), detoxifying enzymatic mechanisms involving a catalase orthologous to KatG (*csal0159*), an iron superoxide dismutase (*csal1861*), the peroxidase OsmC (*csal0037)*, a 1-Cys peroxiredoxin (*csal0179*), and an alkyl-hydroperoxide reductase (*csal0321*) in *C. salexigens* under saline stress has been made by Salvador et al. (2018). The above-mentioned results led us to investigate a possible salt-induced cross-protection mechanism against oxidative stress.

### Glycine Betain Plays an Osmolarity Regulation Role in ANJ207

**FIGURE 6:**
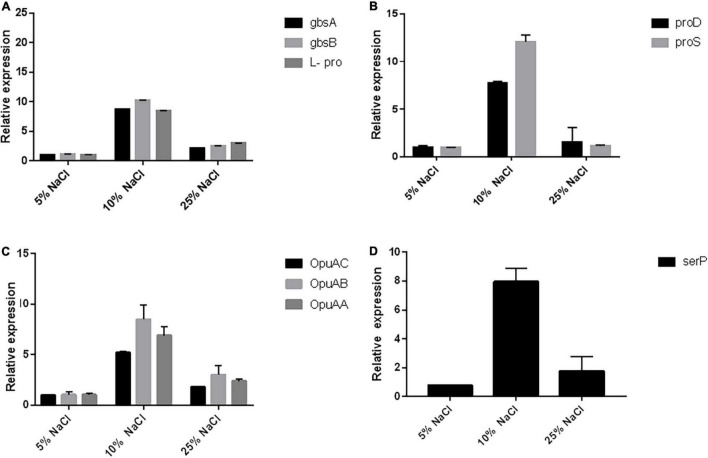
RT-PCR reveals the upregulation of the pathways related to osmotic stress. **(A)** Represents the qRT-PCR of the genes responsible for the glycine, betain, and proline biosynthesis (gbcA, gbcB, and L-pro). Similarly, **(B)** represents the qRT-PCR genes involved in the biosynthesis of L-hydroxyproline (proD and proS), **(C)** represents the qRT-PCR of genes related to glycine betain transporter subunits (OpuAC, OpuAA, and OpuAB), and **(D)** represents qRT-PCR of the genes involved in the stress response proteolysis gene for periplasmic membrane stress sensing (serP).

Most of the osmoprotectants, particularly glycine betain (GB), are widely available in the environment and can be easily accumulated in microorganisms and plants in response to salt stress. Most of the microorganisms accumulate the GB either from the environment through various transport systems like the betaine/choline/carnitine transporter (BCCT) family and the ABC transporters located on cellular membranes, or through the *de novo* synthesis *via* the choline oxidation pathway, with betaine aldehyde as the intermediate (Chen et al., 2010). The gbcA and gbcB genes have been proven to be essential for the GB catabolism *via* the gene disruption strategy in *P. aeruginosa* (Wargo et al., 2008), and their overexpression was shown to be sufficient to reduce the intracellular GB pool (Fitzsimmons et al., 2012). These two genes have been proven to encode the dioxygenase enzyme that can remove the methyl group from the GB and produce dimethylglycine and formaldehyde which may further help in osmolarity regulation (Wargo et al., 2008). We checked the expression of *gbcA* and *gbcB* by RT-PCR and observed ∼10-fold increase at 10% NaCl concentration when compared to the 5% salt concentration, while there was a further reduction in the expression at 25% salt concentration (Figure 6A). The GB plays an important role in *B. subtilis* in osmoregulation, as it can be both synthesized and imported through high-affinity transport systems (Hoffmann et al., 2013).

The *de novo* synthesis of the GB mainly depends on the uptake of the precursor molecule choline through OpuA, OpuB, and OpuC transporters (Shao et al., 2017), which then undergoes a two-step oxidation reaction catalyzed by the orthologous genes of gcbA and gcbB enzymes to produce glycine betaine (Daughtry et al., 2012). The ABC transporters OpuA, OpuC, and OpuD mediate the import of glycine betaine (Shao et al., 2017). OpuA is a high-affinity GB-binding protein tethered to the membrane *via* a lipid anchor and consists of an ATPase OpuAA, the integral membrane protein OpuAB, and the solute receptor OpuAC (Calderón et al., 2004; Lee et al., 2005). The OpuA gene cluster (opuAA, opuAB, and opuAC) in *B. subtilis* is inducible in response to salt stress (Calderón et al., 2004). The *Lactococcus lactis* has also been reported with the osmotically controlled transport activity of OpuA (Rosche et al., 1995). We also checked the osmotic control of opuA expression in response to osmotic stress conditions in *Chromohalobacter salexigens* ANJ207 at three different concentrations of salt (5, 10, and 25% NaCl) and found ∼10-fold increase in the expression of OpuA transport system at 10% salt concentration when compared to 5% salt concentration. Further increase in the salt concentration led to a decrease in the expression, but still, the expression was ∼5-fold high when compared to that observed in 5% salt solution (Figure 6C).

We also checked the expression of the genes involved in the biosynthetic pathway of proline biosynthesis. The expression of the two enzymes delta-1-pyrroline-4-hydroxy-2 carboxylate deaminase (proD) and gamma-glutamyl phosphate reductase (proS) was checked. These two enzymes catalyze the initial stages of the L-hydroxyproline synthesis. We observed the increased expression of these two genes at higher salt concentrations up to 10% NaCl, but further increase in the concentration led to a reduction in the expression (Figure 6B). We also checked the expression of one stress response proteolysis gene *serP* (serine protease) (Figure 6D). The presence of genes for various compatible solutes indicated that a coordinated contribution by various compatible solutes might be responsible for the salinity adaptation of ANJ207. We checked the expression of this gene and found an eight fold increase in the expression at 10% salt concentration. The molecular profiling of osmoregulatory genes showed the presence of genes responsible for the biosynthesis of glycine, betaine, and proline (gbcA, gbcB cluster, proD, and proD) and transporters for the glycine, betaine, choline, and proline (ProP, OpuAC, OpuAA, and OpuAB) uptake along with the stress response proteolysis gene for periplasmic membrane stress sensing (serP, serine protease).

## Conclusion

The present study explores the transcriptome of plant growth-promoting bacterium, *C. salexigens* ANJ207, under different salt concentrations and identified several genes associated with osmotic stress adaptation and mechanisms involved in the production of secondary metabolites under saline conditions. The research findings have shown that at lower salt concentrations, only 92 genes were upregulated, while at higher concentrations of the salt, more than 1,500 genes were upregulated. Furthermore, it has been noticed that a rise in salt concentration not only induces the genes related to osmolarity regulation but also induces the genes related to protein folding and oxidative stress. The glycine betaine was found to be important in the osmolarity regulation in ANJ207. The gene related to GB biosynthesis and the genes for the transport of the GB were also upregulated. These findings will further help to unravel the complex biological mechanisms involved in osmotic stress adaptation and pathways involved in the production of secondary metabolites under saline conditions. Harnessing the potential of *C. salexigens* ANJ207 and its secondary metabolites for the development of novel bioinoculant can be one of the prospective solutions to overcome soil salinity problems in near future.

## Data Availability Statement

The datasets presented in this study can be found in online repositories. The names of the repository/repositories and accession number(s) can be found below: https://www.ncbi.nlm.nih.gov/, MZZK00000000.

## Author Contributions

AlSr and PK conceived the idea. AlSr, RS, and APB did the formal analysis, validation of the data, and writing—draft. AS, JY, AlSi, PT, and AnSr performed the experiments. PK, HC, and AnSa gave critical inputs. All authors have read and approved the manuscript.

## Conflict of Interest

The authors declare that the research was conducted in the absence of any commercial or financial relationships that could be construed as a potential conflict of interest.

## Publisher’s Note

All claims expressed in this article are solely those of the authors and do not necessarily represent those of their affiliated organizations, or those of the publisher, the editors and the reviewers. Any product that may be evaluated in this article, or claim that may be made by its manufacturer, is not guaranteed or endorsed by the publisher.
