# Supplementary material for: Transcriptome Analysis to Understand Salt Stress Regulation Mechanism of Chromohalobacter salexigens ANJ207
Source: Front Microbiol. 2022 Jun 30;13:909276. doi: 10.3389/fmicb.2022.909276 (PMC9279137; doi:10.3389/fmicb.2022.909276)
Supplement: Supplementary file 1 [file Data_Sheet_1.docx]

**Transcriptome analysis to understand salt stress regulation mechanism of *Chromohalobacter salexigens* ANJ207**

Alok Kumar Srivastava^1#^*, Ruchi Srivastava^1#^, Anjney Sharma^1^, Akhilendra Pratap Singh^1^, Jagriti Yadav^1^, Alok Kumar Singh^1^, Praveen Kumar Tiwari^1^, Anchal Kumar Srivatava^1^, Hillol Chakdar^1^, Prem Lal Kashyap^2^ and Anil K Saxena^1^

1. ICAR-National Bureau of Agriculturally Important Microorganisms, Mau-275103, (U.P.) India
2. ICAR-Indian Institute of Wheat and Barley Research, Karnal-132001, India

*Correspondence:

Alok Kumar Srivastava

Principal Scientist,

Genomics Sequencing laboratory,

ICAR- National Bureau of Agriculturally Important Microorganisms (NBAIM), Maunath Bhanjan-275103, UP, India, e-mail: aloksrivastva@gmail.com

Prem Lal Kashyap

Scientist (Plant Pathology)

ICAR-Indian Institute of Wheat & Barley Research (IIWBR)

Karnal-132001, India

Phone: 0184-2267495

Fax: 0184-2267390

Email: [plkashyap@gmail.com](mailto:plkashyap@gmail.com); [Prem.Kashyap@icar.gov.in](mailto:Prem.Kashyap@icar.gov.in)

# Authors have contributed equally

**Supplementary figure**

**Figure S1:** Statistics of the distribution of the FPKM values. The cleaned reads were aligned to the assembled transcriptome (length >= 200bp) using Bowtie2 program and expression value (FPKM) distribution is shown.

**Figure S2: E value distribution and similarity score.** The assembled transcripts were compared with uniprot database using BLASTX program with Evalue cutoff of 10^-3^. The best BLASTX hit based on query coverage, identity, similarity score and description of each transcript was filtered out using in-house pipeline.


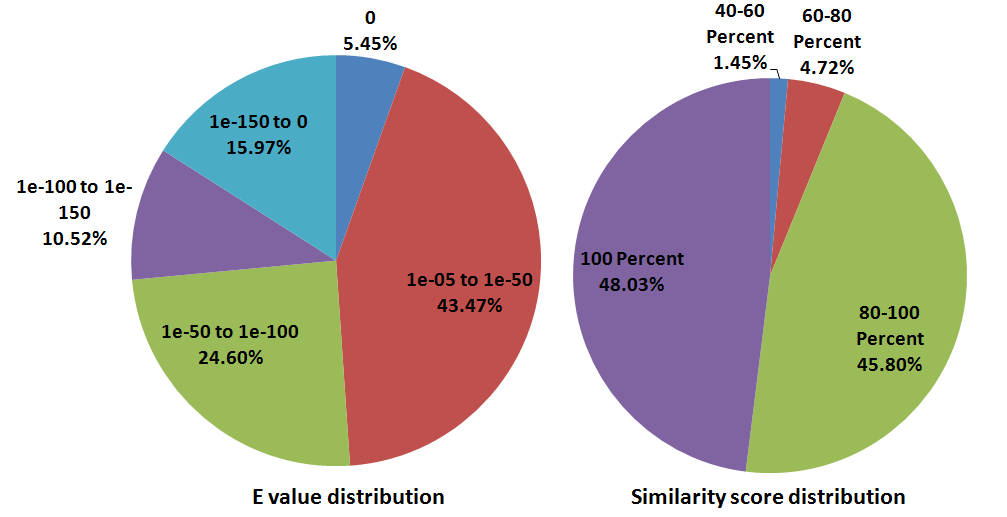


**Figure S3:** Distribution of top organisms corresponding to best BLASTX hits.

**
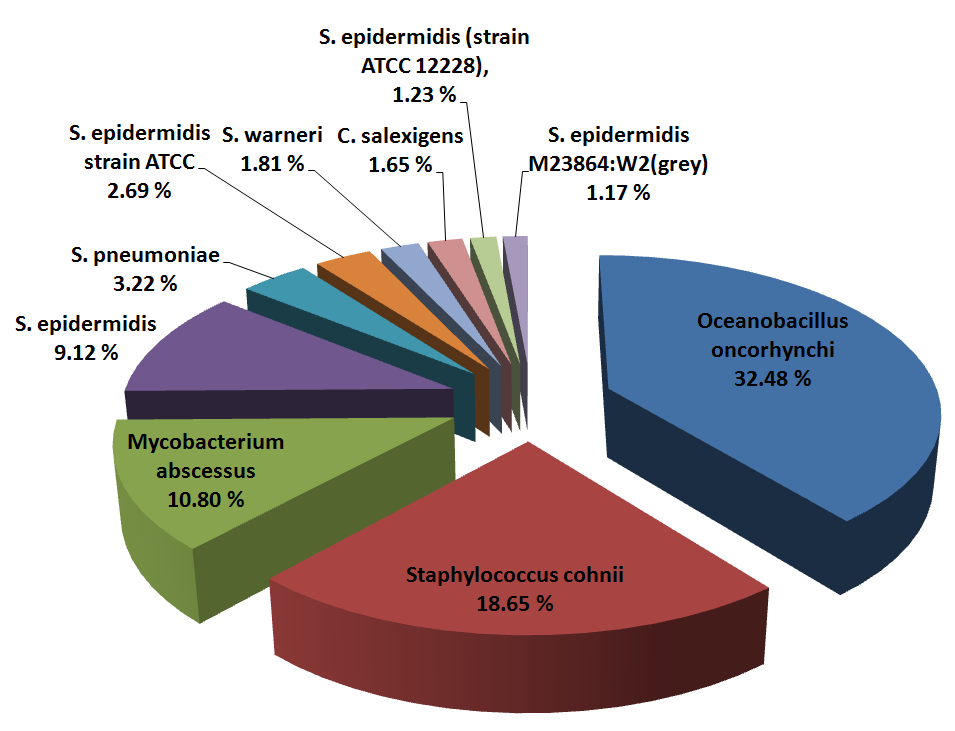
**

**Supplementary table**

**Supplementary table S1:** Raw read summary.

| **Sample Name** | **Number of paired-end reads** | **Number of bases (MBs)** | **GC%** | **Read length (bp)** |
| --- | --- | --- | --- | --- |
| C1 | 3,88,57,852 | 3,885.78 | 35.45 | 100 |
| C2 | 3,57,51,018 | 3,575.10 | 35.36 | 100 |
| C3 | 4,46,95,124 | 4,469.52 | 35.3 | 100 |

**Supplementary table S2:** Clean read statistics.

| **Sample_Id** | **Number of paired-end reads** | **Number of bases (Mb)** | **GC%** |
| --- | --- | --- | --- |
| C1 | 3,74,40,398 | 3,207.19 | 35.97 |
| C2 | 3,44,29,792 | 2,956.83 | 35.895 |
| C3 | 4,29,32,742 | 3,688.10 | 35.86 |

**Supplementary table S3:** Assembled transcripts summary.

| **Assembly** | **C1, C2 and C3** |
| --- | --- |
| Number of Transcripts | 8,936 |
| Smallest | 201 |
| Largest | 1,56,802 |
| Number of Bases | 1,22,12,925 |
| Mean Length | 1,366.71 |
| Transcripts under 200bp | 0 |
| Transcripts over 1000bp | 2,161 |
| Transcripts over 10000bp | 135 |
| n90 | 413 |
| n70 | 1,509 |
| n50 | 4,109 |
| n30 | 24,981 |
| n10 | 78,552 |

**Supplementary table S4:** Summary of alignment.

| **Sample Name** | **Percentage of alignment** |
| --- | --- |
| C1 | 85.50 |
| C2 | 84.35 |
| C3 | 94.10 |

**Supplementary table S5: Summary of BLAST-X.** The assembled transcripts were compared with uniprot database using BLASTX program with E value cutoff of 10^-3^. The best BLASTX hit based on query coverage, identity, similarity score and description of each transcript was filtered out using in-house pipeline.

| **Sample Name** | **C1, C2 and C3** |
| --- | --- |
| Total Number of Transcripts | 8,936 |
| Number of transcripts with significant BLASTX hit | 8,649 |

**Supplementary table S6:** Differentially expressed genes related to protein folding and oxidative stress at different salt concentration.

| **Contig_ID** | **FC** | **logFC** | **PValue** | **gene_name** | **protein_name** |
| --- | --- | --- | --- | --- | --- |
| **C1 v C2** | | | | | |
| TRINITY_DN1222_c0_g2_i1 | 2.853661 | 1.512813807 | 0.000748 | OsmC | OsmC-like protein |
| TRINITY_DN4787_c0_g1_i1 | 2.329412 | 1.219965756 | 0.005421 | SAMEA3109313_01274 | Universal stress protein family |
| **C1 vs C3** | | | | | |
| TRINITY_DN1222_c0_g2_i1 | 2.361154 | 1.239492341 | 0.005587 | SAMEA3109313_01750 | OsmC-like protein |
| TRINITY_DN1227_c0_g2_i2 | 5.169248 | 2.369954511 | 7.26E-05 | SAMEA3109313_00401 | Hsp90-like protein |
| TRINITY_DN1396_c1_g2_i1 | 2.759476 | 1.464394161 | 0.000506 | katA SAMEA3109313_00577 | Catalase |
| TRINITY_DN1396_c1_g3_i1 | 7.739205 | 2.952185375 | 0.002365 | katA | Catalase |
| TRINITY_DN1415_c1_g15_i1 | 12.20717 | 3.609657071 | 1.99E-15 | katG Csal_0159 | Catalase-peroxidase; CP; Peroxidase/catalase |
| TRINITY_DN2895_c0_g1_i1 | 5.564887 | 2.476352501 | 2.37E-08 | katA_2 BN997_02407 | Catalase |
| TRINITY_DN3987_c0_g1_i1 | 3.725702 | 1.89751227 | 0.000568 | BN997_04450 | Universal stress protein |
| TRINITY_DN701_c0_g1_i1 | 10.31944 | 3.36729274 | 0.005282 | nhaX BN997_02773 | Stress response protein NhaX |
| TRINITY_DN7049_c0_g1_i1 | 3.141495 | 1.651451332 | 0.011463 | SAMEA3109313_01849 | Nitroreductase |
| **C2 vs C3** | | | | | |
| TRINITY_DN1227_c0_g2_i2 | 2.603427 | 1.38041172 | 0.011601 | SAMEA3109313_00401 | Hsp90-like protein |
| TRINITY_DN1396_c1_g2_i1 | 2.933632 | 1.552687747 | 0.000236 | katA SAMEA3109313_00577 | Catalase |
| TRINITY_DN1396_c1_g3_i1 | 7.133038 | 2.834516742 | 0.006966 | katA | Catalase |
| TRINITY_DN1415_c1_g15_i1 | 12.65907 | 3.662099556 | 9.16E-16 | katG Csal_0159 | Catalase-peroxidase; CP; Peroxidase/catalase |
| TRINITY_DN2895_c0_g1_i1 | 15.71063 | 3.973669092 | 2.41E-16 | katA_2 BN997_02407 | Catalase |
| TRINITY_DN3987_c0_g1_i1 | 15.1041 | 3.916868766 | 2.41E-08 | BN997_04450 | Universal stress protein |
| TRINITY_DN571_c0_g1_i1 | 11.55549 | 3.530506129 | 0.002134 | TB15 SAMEA2071560_07346 | Universal stress protein |
| TRINITY_DN6171_c0_g1_i1 | 8.859257 | 3.147185745 | 0.013412 | SAMEA2071560_05841 | Activator of Hsp90 ATPase homolog 1-like protein |
| TRINITY_DN701_c0_g1_i1 | 9.533315 | 3.252977908 | 0.00839 | nhaX BN997_02773 | Stress response protein NhaX |

**Supplementary table S7: G**enes related to glycine, betaine synthesis and their transport as well as genes related to the oxidative stress and their fold change.

| Contig_ID | logFC | PValue | evalue | gene_name | protein_name |
| --- | --- | --- | --- | --- | --- |
| TRINITY_DN3165_c0_g1_i1 | 3.367293 | 0.005282 | 4.10E-79 | ATY33_09630 | Proline dehydrogenase |
| TRINITY_DN705_c0_g1_i1 | 1.786685 | 2.58E-05 | 8.70E-245 | UF66_2618 | L-Proline/Glycine betaine transporter ProP |
| TRINITY_DN1405_c1_g1_i1 | 3.574632 | 3.32E-15 | 0 | gcvP Csal_1811 | Glycine dehydrogenase |
| TRINITY_DN2539_c0_g1_i1 | 1.638084 | 0.000203 | 2.60E-268 | opuD | Glycine betaine transporter OpuD |
| TRINITY_DN2543_c0_g1_i1 | 1.335068 | 0.0075 | 7.80E-199 | CIL03_04735 | Glycine/betaine ABC transporter permease |
| TRINITY_DN317_c0_g1_i1 | 1.268305 | 0.011019 | 1.30E-269 | opuCC | Glycine/betaine ABC transporter periplasmic protein |
| TRINITY_DN6807_c0_g1_i1 | 2.925936 | 0.001197 | 6.80E-80 | dfrA BN997_03626 | Dihydrofolate reductase |
| TRINITY_DN1400_c1_g7_i1 | 1.998823 | 3.50E-05 | 3.10E-232 | gamma-BBH | Gamma-butyrobetaine hydroxylase |
| TRINITY_DN3165_c0_g1_i1 | 3.367293 | 0.005282 | 4.10E-79 | ATY33_09630 | Proline dehydrogenase |
| TRINITY_DN1400_c1_g7_i1 | 1.998823 | 3.50E-05 | 3.10E-232 | gamma-BBH | Gamma-butyrobetaine hydroxylase |
| TRINITY_DN431_c0_g1_i1 | 2.215445 | 0.000513 | 7.50E-154 | trxB_2 BN997_02679 | Thioredoxin reductase |
| TRINITY_DN5732_c0_g1_i1 | 1.819227 | 0.000721 | 2.60E-55 | trxA | Thioredoxin |
| TRINITY_DN5741_c0_g1_i1 | 2.252342 | 0.009733 | 9.90E-56 | ydbP BN997_02367 | Thioredoxin-like protein YdbP |
| TRINITY_DN1149_c0_g1_i1 | 1.837229 | 0.001052 | 1.60E-195 | yhhX BN997_00195 | Putative oxidoreductase YhhX |
| TRINITY_DN1388_c1_g1_i10 | 2.703731 | 1.14E-05 | 3.30E-171 | ydhF BN997_00911 | Oxidoreductase YdhF |
| TRINITY_DN2057_c0_g1_i1 | 2.632727 | 0.007865 | 6.10E-121 | yhfP BN997_02748 | Putative quinone oxidoreductase YhfP |
| TRINITY_DN2515_c0_g1_i1 | 1.944805 | 0.000311 | 1.90E-106 | SAMEA3109313_02298 | Oxidoreductase ylbE |
| TRINITY_DN2496_c0_g1_i1 | 1.441699 | 0.00226 | 4.70E-158 | msrR | Peptide methionine sulfoxide reductase |
| TRINITY_DN1409_c0_g4_i4 | 3.986491 | 6.78E-18 | 0 | Csal_0292 | 4-hydroxyphenylpyruvate dioxygenase |
| TRINITY_DN6910_c0_g1_i1 | 1.482737 | 0.006877 | 5.40E-117 | sodA_2 BN997_04089 | Superoxide dismutase |
| TRINITY_DN1415_c1_g15_i1 | 3.609657 | 1.99E-15 | 0 | katG Csal_0159 | Peroxidase/catalase |
| TRINITY_DN1276_c0_g3_i1 | 1.930881 | 5.65E-06 | 2.10E-285 | ahpF | Alkyl hydroperoxide reductase |
| TRINITY_DN3030_c0_g1_i1 | 3.033018 | 0.013412 | 3.90E-84 | bcp BN997_03069 | Putative peroxiredoxin bcp |
| TRINITY_DN985_c1_g1_i1 | 2.650288 | 0.003149 | 3.30E-41 | SAMEA2071560_07188 | Ferredoxin |
| TRINITY_DN4619_c0_g1_i1 | 1.312408 | 0.012577 | 0 | cobT SAMEA3109313_00955 | Nitric oxide reductase activation protein NorD |
| TRINITY_DN5838_c0_g1_i1 | 6.458772 | 0.002435 | 2.60E-57 | norM BN997_03395 | NorM |
